# Looking for Lepidic Component inside Invasive Adenocarcinomas Appearing as CT Solid Solitary Pulmonary Nodules (SPNs): CT Morpho-Densitometric Features and 18-FDG PET Findings

**DOI:** 10.1155/2019/7683648

**Published:** 2019-01-13

**Authors:** Alfonso Reginelli, Raffaella Capasso, Mario Petrillo, Claudia Rossi, Pierluigi Faella, Roberta Grassi, Maria Paola Belfiore, Giovanni Rossi, Maurizio Muto, Pietro Muto, Alfonso Fiorello, Nicola Serra, Rita Nizzoli, Massimo De Filippo, Salvatore Cappabianca, Gianpaolo Carrafiello, Luca Brunese, Antonio Rotondo

**Affiliations:** ^1^Department of Internal Clinical and Experimental Medicine and Surgery, University of Campania “Luigi Vanvitelli”, 80138 Naples, Italy; ^2^Department of Medicine and Health Sciences, University of Molise, 86100 Campobasso, Italy; ^3^Unit of Radiation Oncology, University Hospital of Florence, 50134 Florence, Italy; ^4^Department of Radiology, V. Monaldi Hospital, Naples, Italy; ^5^Thoracic Surgery Unit, University of Campania “Luigi Vanvitelli”, 80138 Naples, Italy; ^6^Oncology Unit, Parma Hospital, University of Parma, 43126 Parma, Italy; ^7^Department of Surgical Sciences, Section of Diagnostic Imaging, Parma Hospital, University of Parma, 43126 Parma, Italy; ^8^Diagnostic and Interventional Radiology Department, ASST Santi Paolo and Carlo-San Paolo Hospital, University of Milan, 20142 Milano, Italy

## Abstract

**Objective:**

To investigate CT morphologic and densitometric features and 18-FDG PET findings of surgically excised lung adenocarcinomas “mixed subtype” with predominant lepidic component, appearing as solid solitary pulmonary nodules (SPNs) on CT scan.

**Materials and Methods:**

Approval for this study was given from each local institutional review board according to its retrospective nature. Nodules pathologically classified as lung adenocarcinoma mixed subtype with bronchioloalveolar otherwise lepidic predominant component, in three different Italian institutions (Napoli; Varese; Parma), were retrospectively selected.

**Results:**

22 patients were identified. The number of SPNs with smooth margins was significantly lower with respect to the number of SPNs with spiculated margins (p: 0.033), radiating spiculations (p: 0.019), and notch sign (p: 0.011). Mean contrast enhancement (CE) was 53.34 HU (min 5.5 HU, max 112 HU); considering 15 HU as cut-off value, CE was positive in 20/22 cases. No significant correlation was found between size and CE. Mean SUVmax was 2.21, ranging from 0.2 up to 7.5 units; considering 2.5 units as cut-off, SUVmax was positive in 7/22 cases. The number of SPNs with positive CE was significantly higher than the number of SPNs with positive SUVmax (p: 0.0005).

**Conclusion:**

CT generally helps in identifying solid SPN suspicious for malignancy but 18-FDG PET may result in false-negative evaluation; when 18-FDG PET findings of a solid SPN are negative even though CT morphology and CE suggest malignancy, radiologist should consider that lepidic component may be present inside the invasive tumor, despite the absence of ground glass.

## 1. Introduction

Lung adenocarcinoma (LA) is the most common histological subtype of lung cancer representing the leading cause of cancer-related death in both men and women throughout the world [1, 2]. When LA presents as a solitary pulmonary nodule (SPN) it is often still in stage I, and the patients often can be cured or have a long survival after proper treatment [3]. Recent data showed that LA histomorphological growth patterns seem to be associated with different prognosis in terms of survival differences [4]. With this background, in 2011 the International Association for the Study of Lung Cancer, American Thoracic Society, and European Respiratory Society (IASLC/ATS/ERS) proposed an International Multidisciplinary Lung Adenocarcinoma Classification [5]. Ever since, invasive LAs are classified by predominant pattern after using comprehensive histologic subtyping with lepidic, acinar, papillary, and solid patterns; micropapillary is added as a new histologic subtype [2, 5]. Moreover, the terms “bronchioloalveolar” (BAC) and “mixed subtype” LA are no longer used: LAs previously classified as “mixed subtype” with predominant nonmucinous BAC component are now stated as “lepidic predominant adenocarcinomas” (LPAs) [5]. Lepidic component is reported to influence the appearance of the SPN on computed tomography (CT) scan [6–10]: lepidic growth appears hazy and nonsolid (ground-glass) while the solid component is partly related to invasive growth of the tumor [5, 11]. Therefore, lepidic growth is less likely to occur in solid SPN when compared with semi-solid and ground-glass nodules [4, 12]. Since the presence of lepidic component is reported to determine a more favorable survival for small solitary resected invasive LAs [6], our aim was to investigate CT morphologic and densitometric features and 18-fluorine-fluorodeoxyglucose (18-FDG) findings of surgically excised LAs previously named “mixed subtype” with predominant BAC component, now stated as lepidic component, appearing as solid SPNs on CT scan.

## 2. Materials and Methods

### 2.1. Population

Approval for this study was given from each local institutional review board according to its retrospective nature. In a search of lung cancer pathological registry databases of three different Italian institutions (Ospedale Monaldi, Azienda dei Colli Napoli; Ospedale di Circolo Fondazione Macchi, Varese; Azienda Ospedaliera Universitaria di Parma, Parma) 1311 surgically excised SPNs were retrospectively identified, from January 2012 to December 2014.

Among all SPNs excised, nodules classified as LA mixed subtype with BAC predominant component were retrospectively selected. Then, for each SPN, picture archiving and communication systems (PACS) were screened for available preoperative multidetector CT examinations and 18-FDG PET-CT studies.

Inclusion criteria for the study were histological diagnosis of LA mixed subtype with predominant BAC otherwise lepidic component, single solid nodule on CT examination with no evidence of malignant satellite nodules and without hilar or mediastinal lymphadenopathy, availability of a recent (< 4 weeks) preoperative 18-FDG PET-CT study.

### 2.2. Imaging and Interpretation

#### 2.2.1. CT Images Analysis

Images were acquired using 3 different 64 rows CT scanners (LightSpeed Volume CT, GE Healthcare, United States; Toshiba Aquilion 64 system, Toshiba Medical Systems, Otawara; Sensation 64, Siemens, Erlangen, Germany) after having obtained patients' informed consent. Unenhanced CT images were acquired in spiral mode and scans were performed at suspended maximal inspiration after appropriate instruction of the patients to minimize breathing artifacts. Contrast enhanced scans were acquired in spiral mode during the portal phase after intravenous administration of 1-2 ml/Kg body weight, limited to 150 mL, of nonionic iodinated contrast medium (CM) at a rate of 2.5-3.5 mL/s, followed by a flush of saline solution at a rate of 2.5 mL/s.

All CT images were evaluated on vendors' CT workstations displaying images on two monitors to view both mediastinal (width, 400 HU; level, 40 HU) and lung (width, 1500 HU; level, -500 HU) window images. Two independent radiologists who were unaware of the clinical 18-FDG PET findings and blind to each other, retrospectively, evaluated all CT examinations to select SNPs which fulfilled the above-mentioned inclusion criteria. Any differences were resolved by consensus; for quantitative variables, the mean value of the two radiologists' measurements was selected. CT images were assessed for the appearance of each nodule to select nodules that appeared solid, evaluating the maximum axial size (Dmax), margins, presence of notch sign and radial spiculations, seat of the lesion, and finally CE. LA nodules were considered solid on CT images if nodules completely obscured the entire lung parenchyma within them [13]. The size of each nodule was assessed according to the longest diameter on the transverse lung window image where the largest nodule dimension appeared. Margins of the nodules were distinguished as smooth, lobulated, irregular, and spiculated, while an irregular indentation of the margin of the nodule, a “concave cut”, was stated as notch [11]. Radial spiculations were defined as lines radiating from the margins of the nodule [14]. Moreover, the presence of fat and/or calcifications inside the nodule and SPN localization in lung segments were noticed. After morphological evaluation, densitometric measurements of SPNs were obtained in both precontrast and postcontrast phases. A region of interest (ROI) was drawn inside each SPN along the equatorial plane, as large as possible in order to minimize noise, but care was taken to avoid partial volume effects and any calcified inclusions, vessels, bronchogram, or inner necrotic areas. To avoid interfusion of air space, the reader magnified the image for display on the viewer and drew ROIs inside the boundary of tumors. SPN attenuation value of enhanced CT scan minus the baseline attenuation on unenhanced CT scan was defined as CE considering 15 HU as cut-off value.

#### 2.2.2. 18-FDG PET-CT Images Analysis

Two types of hybrid PET-CT scanners were used (Biograph 16; Siemens, Erlangen, Germany; GE, Milwaukee, USA). In all cases, 18-FDG PET acquisition was performed from feet to head 60 minutes after intravenous administration of 18F-FDG (4.5 MBq/kg). Emission scan bed time was 3 min, resulting in a total PET scan time of approximately 20-25 min.

Hybrid 18-FDG PET-CT images were reconstructed on each specific workstation. The referring experienced interpreters of each institution, who were unaware of clinical and pathologic results, evaluated 18-FDG PET-CT images by a semiquantitative analysis of 18-FDG uptake based on a volume of interest to calculate the maximum standard uptake value (SUVmax) considering 2.5 units as cut-off value.

### 2.3. Statistical Analysis

The variables considered for morphologic analysis were size (Dmax), type of margins, presence of notch sign, and radial spiculations; densitometric evaluation was based on CE value in portal phase while metabolic assessment concerned with SUVmax.

Student T-test was used for the comparison of two means applied to small populations samples (i.e., mean Dmax, margins, mean CE, and mean SUVmax). Pearson correlation coefficient was used to measure the strength of a linear association between two variables (i.e., maximum diameter, types of margins, CE, and SUVmax). McNemar's exact test was used to compare paired proportions (i.e., the percentage of SPNs with positive CE and the percentage of SPNs with positive SUV, the percentage of SPNs with spiculated margins, and the percentage of SPNs with smooth margins).

Continuous and categorical variables were analyzed (Student T-test, Pearson correlation coefficient, and McNemar's exact test) with Matlab statistical toolbox version 2008 (MathWorks, Natick, MA, USA) for Windows at 32 bit. A p-value < 0.05 was considered significant.

## 3. Results and Discussion

### 3.1. Results

22 patients (17 males and 5 females) with a mean age of 71.09±9.16 years were finally identified, who fulfilled the inclusion criteria. CT morpho-densitometric features and SUVmax values of the 22 SPNs evaluated are reported in Table 1.

Main significant results of imaging analysis are summarized in Table 2, expressed as the mean value and standard deviation (SD) for continuous variables and as percentage for categorical variables.

Among the 22 SPNs selected, mean maximum diameter was 15.45 mm (range 7–26 mm). Most (9/22) of SPNs presented spiculated margins, while margins were lobulated in 6/22 cases, irregular in 6/22 cases, and smooth in 2/22 cases. The notch sign was present in 10 nodules and radial spicules were observed in 9 cases.

The number of SPNs with smooth margins were significantly lower with respect to the number of SPNs with spiculated margins (p: 0.033), radiating spiculations (p: 0.019), and notch sign (p: 0.011).

All solid nodules did not contain any calcifications or fat.

SPNs were peripherally located as follows: 3 in the apical segment of right upper lobe, 5 in the apico-dorsal segment of the left upper lobe, 3 in the dorsal segment of the right upper lobe, 1 in the middle lobe, 1 in the apical segment of the right lower lobe, 3 in the apical segment of the left lower lobe, 1 in the lingula, and 5 in the posterior basal segment of the right lower lobe.

Mean CE was 53.34 HU (min 5.5 HU, max 112 HU); considering 15 HU as cut-off value, CE was positive in 20/22 cases. No significant correlation was found between size and CE.

Mean SUVmax was 2.21, ranging from 0.2 up to 7.5 units; considering 2.5 units as cut-off, SUVmax was positive in 7/22 cases.

The number of SPNs with positive CE was significantly higher than the number of SPNs with positive SUVmax (p: 0.0005).

SUVmax showed statistically significant correlations with Dmax (R:0.501) and with lobulated margins (R:0.455); in particular, SPNs with positive SUVmax presented diameters statistically significant (p: 0.038) greater than SPNs with negative SUVmax.

### 3.2. Discussion

SPNs are frequently encountered on chest imaging and represent an important diagnostic challenge because their radiologic characterization is very complex [15, 16]. The evaluation of morphologic features is recognized to be a useful tool to assess the likelihood of malignancy, although there is a considerable overlap between benign and malignant morphology in many cases [17, 18]. Moreover, despite CT technological advances, determining the etiology of a lung nodule without invasive approach remains problematic and the current “gold standard” for diagnosing pulmonary nodules still remains pathology, requiring samples obtained either surgically or by percutaneous biopsy [18–20]. The employment of 18-FDG PET as a diagnostic tool could reduce the number of unnecessary biopsies or thoracotomies on benign SPNs; however it could also show false negative results for lesions smaller than 2 cm and for well-differentiated invasive LAs (i.e., with lepidic component) having low metabolic rate [12, 18].

To predict LA histological subtype and then patient prognosis by using imaging tools despite invasive pathological scoring system may be very attractive. In this context, we aimed to investigate if the presence of lepidic component inside LAs appearing as solid SPNs on CT images could affect their morphological, CE, and SUVmax features.

#### 3.2.1. CT Morpho-Densitometric Features

In our study were included LAs with histopathologically proven predominant lepidic component which appeared as solid SPNs on CT images.

It has been well demonstrated that CT attenuation of lung lesions closely reflects the proportion of histological components; the invasive growth characteristically appears solid and the noninvasive lepidic growth is hazy and nonsolid [9, 11]. According to this, a recent study reported that lepidic growth is significantly less likely to occur in solid tumors as determined by CT (9,6%) when compared with semi-solid (17,7%) and ground-glass opacity (67,5%) tumors [4]. However, a solid appearance on CT can reflect a collapse of the alveolar wall, fibrosis, other than proliferation of invasive tumor cells [21, 22]. These considerations account for the small number of solid SPNs of LA with predominant lepidic component we found; however, the number of our patients is of the same order of size of LPAs described in several reports [2–4, 23, 24]. Although it has been reported that semi-solid or nonsolid nodules are more likely to be malignant than solid ones [13, 16], in our study all solid SPNs were lung cancer. An important aspect of LAs is that, for SPNs, the size of the invasive component is an independent predictor of survival [11]. Furthermore, disease-free survival correlates with solid tumor size but not with “whole tumor size” that includes a nonsolid (ground-glass) component [10, 11]. So, the solid area seems to be a better marker for prognosis prediction compared with the whole nodule diameter [25]. In our study, being all nodules solid on CT images, solid diameter corresponded to the whole nodule diameter. Lesion size is a recognized valid predictor of malignancy: nodules with diameter less than 5 mm, 5 to 10 mm, and greater than 2 cm, are associated with malignancy rates of less than 1%, 6-28%, and 64-82%, respectively [20]. In our study, none of the SPNs had diameter less than 5 mm, while 5 NPSs were greater than 20 mm.

Although it is not possible to differentiate various histological subtypes of LA distinctively at CT according to their appearances [26], it has been reported that margin configuration is associated with distinct histopathological LA growth patterns [4]. Recently, LPAs have been reported to have no predominant margin pattern and this assumption is in agreement with our findings: margins of our SPNs ranged from smooth to spiculated. Lederlin et al. observed that lepidic growth was associated with mixed, irregular, margins (27,4%) more frequently than spiculated (10.4%) or smooth ones (5.4%) [4]. As our study, smooth margins were significantly the less frequent margin type observed (9.09%) also, while spiculated margins, although they represented the majority of cases (40.91%), did not reach a significant statistical predominance. SPNs with irregular, spiculated margins or lobulated contours, are typically associated with malignancy [18]. Smooth margins, however, are not diagnostic of benignity and do not exclude malignity, as up to one-third of malignant nodules have well-defined margins [17]. Edge characteristics indicative of malignancy and correlated with a desmoplastic response in the nodule include also the presence of spicules radiating from the nodule, often described as “sunburst” or “corona radiata” appearance [27]. We noticed sunburst in 9/22 SPNs, while 10/22 SPNs showed the “notch sign": it is reported that notching or umbilication of any portion of the border of a spherical nodule should cause concern for malignancy [28]. In a recent report, notch sign in small (<20 mm) solid-density LA appeared to be a somewhat unfavorable prognostic sign [29]. This notch pattern probably could correspond to the irregular edge of the invasive component shown at the histology [11, 30].

In our study, SPNs did not show a prevalent involvement of a single lung segment but were all located in peripheral parenchyma. However, SPN location alone cannot be used as an independent predictor of malignancy and AC histological subtype [16].

Although several studies reported some associations between tumor CE and grading, intratumoral fibrosis, and angiogenic activity [31–33], to the best of our knowledge, the correlation between CE and AC histologic subtypes according to IASLC/ATS/ERS classification has not yet been investigated. Contrast-enhanced CT improves accuracy of benign versus malignant differentiation of SPNs [19]. Nodule behavior after contrast material administration is sensitive but not specific for malignancy; however nodule enhancement of less than 15 HU is strongly indicative of benignity [16, 18, 34–37]. Higher accuracy is reported for dynamic enhancement evaluation on helical CT (HDCT), analyzing combined wash-in (WI) and washout (WO) characteristics [18, 38]. In our study, given the retrospective assessment of CT exams, we considered only one scan acquired in portal phase after contrast medium administration. In this way, we observed a CE < 15 UH in 2 patients only. Typically, peak nodular enhancement is appreciated with a multiphase dynamic CT scan among 40 to 180 s after IV contrast medium injection [39], then we did not obtain the peak of enhancement, but only a single value of attenuation to compare with the baseline one. This approach could explain why we had 2 CE false negative cases: with more CT acquisitions at different times probably we could observe these nodules enhance more than 15 HU. However, also if CE less than 15 HU indicates benignity [35], these nodules showed other characteristics of suspicious malignancy such as lobulated or spiculated margins and/or SUVmax > 2,5 (Table 1). According to this, it has been reported that the evaluation of solid SPNs by combined analysis of CE values and morphologic features through CT scans can provide 92% sensitivity and 79% specificity [39]. We did not find a significant correlation between CE and size; however positive CE was statistically significant more frequent than morphologic features of malignancy (spiculated or lobulated margins, sunburst and notch sign) (Table 2).

#### 3.2.2. 18-FDG PET-CT Metabolic Findings

18-FDG PET-CT is an essential tool in current lung cancer diagnostic practice: it is not only useful for staging, evaluating therapeutic effects, but it is also useful for differentiating localized pulmonary lesions [10, 12, 40]. SUVmax higher than 2.5 yields likelihood for malignancy [16], although certain neoplasms, such as carcinoid, formerly named BAC and well-differentiated LAs, can have a low metabolic rate that may result in false-negative examinations [18, 41]. Indeed, SUVmax is strongly correlated with pathologic score [2, 10, 42]. It has been described that 18-FDG PET is falsely negative in around 50% of patients with BAC, or adenocarcinoma in situ (AIS) [18]. Even though 18-FDG PET scan findings may be negative, these nonsolid histological type cancers can generally be recognized based on ground-glass findings at CT [12, 14]. However, in clinical practice, lung cancers that do not show ground-glass opacity at CT can also show false-negative 18-FDG PET findings, as in our study [12]. Furthermore, besides histopathological subtype, another primary factor influencing 18-FDG PET findings is the lesion size [12, 43]; this is in agreement with our results that showed a statistically significant (p=0.0155) correlation between lesion size and SUVmax (R=0.501).

Negative 18-FDG PET results for nodules smaller than 1 cm, particularly <7 mm, do not confidently exclude malignancy [16, 18, 44]. In our study, all SPNs were ≥ 7 mm, and SUVmax < 2.5 was observed in 15 cases (68,18%) with a mean Dmax of 14.27 mm (Table 2).

The remaining 7 SPNs presented a SUVmax ≥ 2.5 and most (4/7) of them showed lobulated margins. Both SUVmax ≥ 2.5 and lobulated margins are typically associated with malignancy [16, 18] and this could explain the correlation we found between these two features (R: 0.455).

Mimae et al. proposed that tumor size ≤ 18 mm and SUVmax ≤ 3.2 indicate a low grade malignant tumor and can histologically predict the presence of lepidic component [45]. According to this, in our study sample most (18/22) of SPNs showed size ≤18 mm and SUVmax ≤ 3.2.

**Figure 1:**
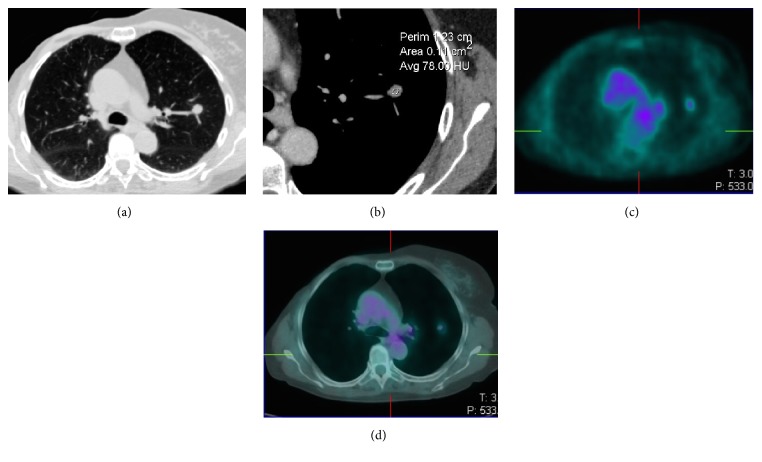
CT (a, b) and 18-FDG PET-CT (c, d) images of a lung adenocarcinoma with lepidic predominant component appearing as a solid nodule with smooth margins (a), showing intense CE (59 HU) despite low SUVmax (1.8).

**Figure 2:**
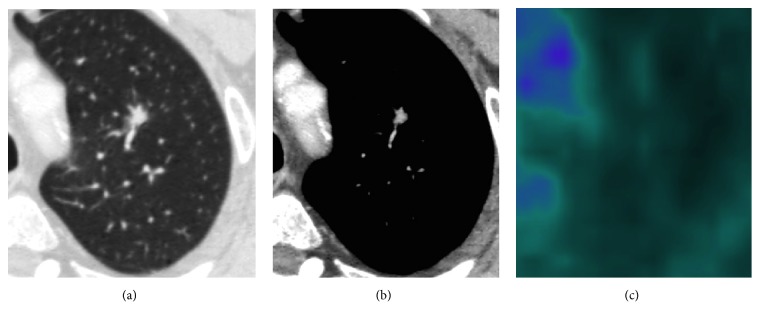
CT (a, b) and 18-FDG PET-CT (c) images of a 18-FDG PET false negative lung adenocarcinoma with lepidic predominant component; the solid nodule presented spiculated margins and notch sign (a), intense CE (88 HU) (b) despite low SUVmax (1.20) at 18-FDG PET examination (c).

**Figure 3:**
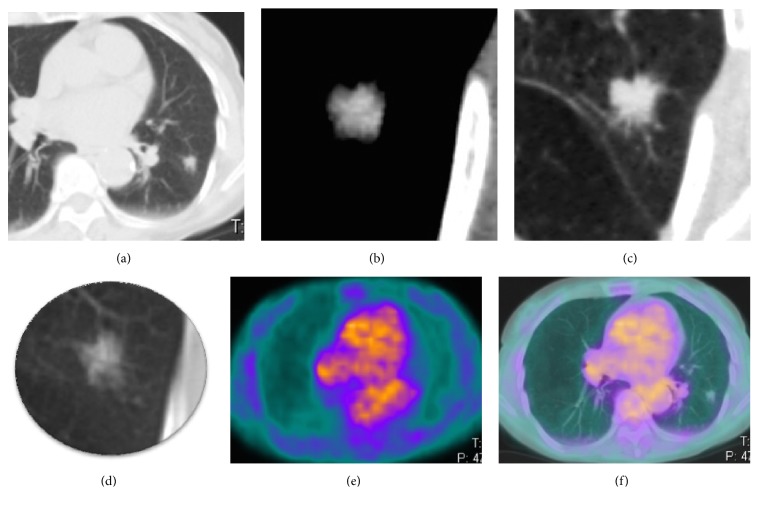
CT (a-d) and 18-FDG PET-CT (e, f) images of a 18-FDG PET false negative lung adenocarcinoma with lepidic predominant component; the solid nodule presented spiculated margins and notch sign (a and b, lung window), intense CE (97 HU) (c, mediastinal window) despite very low SUVmax (0.2) at 18-FDG PET examination (e, f); 1 year before the same nodule appeared as a ground-glass opacity on CT scan (d).

Considering SPNs with SUVmax <2.5, almost all of them (14/15) presented irregular/spiculated margins and/or sunburst, morphologic findings that suggested their malignancy (Table 1). Only 1 nodule with negative SUVmax had smooth margins, but it presented also CE > 15 HU (Figure 1). Then, beyond morphology, CE was positive (≥ 15 HU) in 93.3% of 18-FDG PET false negative SPNs (Figures 2 and 3). Only 1/15 SPN (diameter 10 mm) presented CE <15 HU; however spiculated margins and sunburst were shown. So, combined analysis of CT morphology and CE allowed us to state all SPNs as malignant lesion while low SUVmax oriented in the majority (68.18%) of cases for well-differentiated cancer.

The results of our investigation are consistent with recent studies which report that while high SUV correlates with high-grade histology (solid and micropapillary), low SUVmax correlates with low-grade histology (adenocarcinoma in situ, minimally invasive adenocarcinoma, and LPA) in the IASLC/ATS/ERS classification, representing also a prognostic element for the stratification of patients in Stage I LA [46, 47].

#### 3.2.3. Limits

This study has several limits mainly due to its retrospective design. CT exam technique was not standardized and histological assessment considered only the predominant histologic pattern without semiquantitative assessment of the percentages of the various histologic components proposed by IASLC/ATS/ERS classification. Then, other less represented patterns could have variably influenced CT and 18-FDG PET-CT findings; however this is a limit of the IASLC/ATS/ERS classification and further study is required to evaluate how minor pattern can influence imaging findings and clinical outcome. Neither a survival analysis nor a prognostic evaluation was performed. Furthermore, because some findings of our study had only small numbers of individuals, we cannot make a definite conclusion because of the small sample size and it is likely that some associations were driven by small numbers in the distribution of the parameters evaluated.

## 4. Conclusions

LA with lepidic component are rarely encountered as solid SPNs on CT scan; they consist of a mixture of invasive component together with noninvasive lepidic growth which determines a wide spectrum of CT morphological features, CE behavior, and SUVmax values. CT generally helps in identifying solid SPN suspicious for malignancy but 18-FDG PET may result in false-negative evaluation. Therefore, when 18-FDG PET findings of a solid SPN are negative even though CT morphology and CE suggest malignancy, radiologist should consider that lepidic component may be present inside the invasive tumor, despite the absence of ground glass, determining its low metabolic rate [12].

## Figures and Tables

**Table 1:** *Study sample*. The table shows the morphologic features, CE (contrast enhancement) and SUVmax of all SPNs grouped according to margin type; +: presence; -: absence.

**MARGINS**	**NOTCH**	**SUNBURST**	**DIAMETER (MM)**	**CE (HU)**	**SUVmax**
**SPICULATED **	+	-	8,00	88	1,20
	+	-	10,00	97	0,20
	-	+	10,00	6	1,60
	-	+	12,00	30	0,80
	+	-	13,00	48	0,40
	-	+	18,00	94	0,8
	+	-	19,00	88	0,40
	+	-	22,00	24	1,70
	+	+	26,00	23	7,5

**IRREGULAR**	-	-	7,00	69	2,4
	-	+	12,00	112	2,60
	+	-	18,00	92	1,5
	+	-	24,00	39	5,6
	-	+	24,00	20	0,60

**LOBULATED**	-	-	9,00	5,5	2,6
	-	-	13,00	67	2,5
	-	-	13,00	21	1,50
	+	+	16,00	67	0,20
	-	-	20,00	23	5,3
	-	+	22,00	57	7,14

**SMOOTH**	-	-	11,00	59	1,80
	+	+	13,00	44	0,30

**Table 2:** *Imaging parameters and significant statistical tests*. The table shows the main results of imaging analysis expressed as the mean value and standard deviation (SD) for continuous variables and as percentage for categorical variables. For statistical tests, a p-value < 0.05 was considered significant.

**Parameters**	**Mean ± SD**	**Correlation**	
Maximum diameter (Dmax)	15.45 ± 2.58	Dmax / SUVmax: R = 0.501	
CE	53.34 ± 31.49	SUVmax/Lobulated: R = 0.455	
SUVmax	2.21 ± 2.15		

**Morphologic parameters**	%	**Hypothesis**	**p-value**

Spiculated margins (SpM)	40.91	Spicules (40.91%) vs. SmM (9.09%)	0.0195 (M)
Lobulated margins (LM)	27.27	SpM (40.91%) vs. SmM (9.09%)	0.0327 (M)
Irregular margins (IM)	27.27	Notch (45.45%) vs. SmM (9.09%)	0.0107 (M)
Smooth margins (SmM)	9.09	CE (90.91%) vs. SUVmax (31.82%)	4.9·10^−4^ (M)
Notch	45.45	CE (90.91%) vs. SpM (40.91%)	1.7·10^−3^ (M)
Spicules	40.91	CE (90.91%) vs. LM (27.27%)	2.6·10^−4^ (M)
**Positive CE (≥ 15 HU)**		CE (90.91%) vs. IM (27.27%)	6.0·10^−5^ (M)
CE +	90.91	CE (90.91%) vs. SmM (9.09%)	4.0·10^−6^ (M)
**Positive SUVmax (≥ 2.5)**		CE (90.91%) vs. Notch (45.45%)	9.8·10^−4^ (M)
SUVmax +	31.82	CE (90.91%) vs. Spicules (40.91%)	1.7·10^−3^ (M)

**Parameters **	**Mean ± SD**	** Hypothesis**	**p-value**

Dmax (SUV ≥ 2.5)	18.86 ± 5.62	Dmax (SUV ≥ 2.5) vs. Dmax (SUV < 2.5)	0.0384 (T)
Dmax (SUV < 2.5)	14.27 ± 4.88		

T: T-Student test; R: Pearson's linear correlation coefficient; M: McNemar's exact test.

## Data Availability

The imaging analysis data used to support the findings of this study are included within the article.
